# Double arterial cannulation for contained rupture of a mycotic innominate artery aneurysm

**DOI:** 10.1016/j.xjtc.2025.07.022

**Published:** 2025-08-06

**Authors:** Étienne Fasolt Richard Corvin Meinert, Rawa Arif, Matthias Karck, Bashar Dib

**Affiliations:** Department of Cardiac Surgery, Heidelberg University Hospital, Heidelberg, Germany

**Figure:**
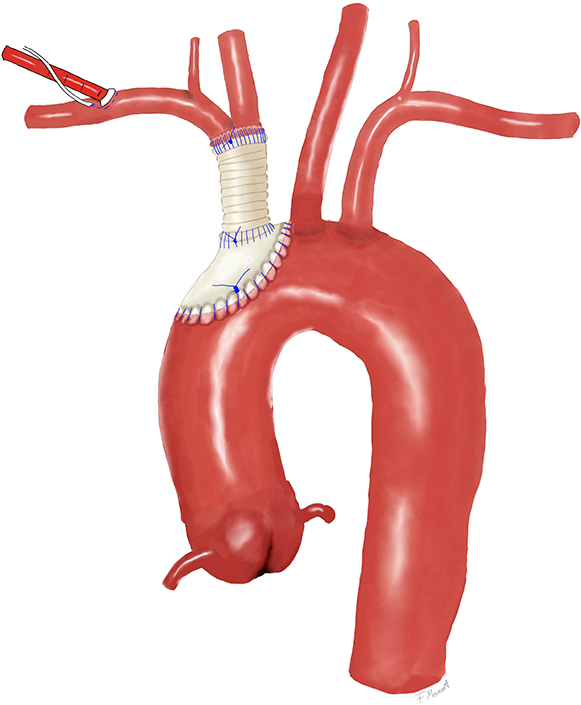
Reconstruction using a 10-mm Dacron graft and a pericardial patch.

**Central Message**

This case shows that prudent cannulation strategies allow for successful treatment of rare and complex pathologies, such as ruptured mycotic aneurysm of the innominate artery.

Mycotic aneurysms of the innominate artery are extremely rare conditions. Double arterial cannulation was used to address a contained rupture of a mycotic innominate artery aneurysm and mediastinitis caused by *Staphylococcus aureus*.

## Case Report

**Figure 1:**
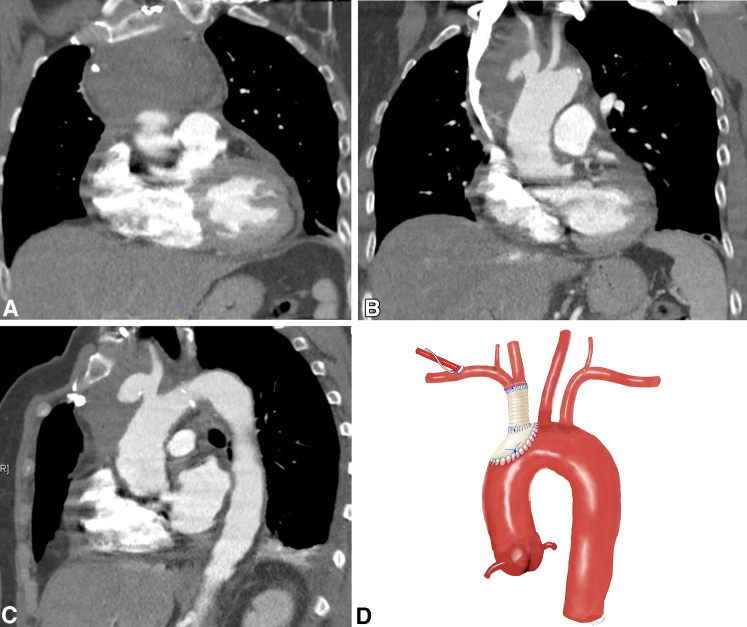
A, Preoperative CT scan showing a large hypodense mass in the anterior mediastinum, coronal plane. B, Preoperative CT scan showing the contained rupture of the mycotic aneurysm of the innominate artery, coronal plane. C, Preoperative CT scan showing the contained rupture of the mycotic aneurysm of the innominate artery, sagittal plane. D, Illustration of the final result of the reconstruction. *CT*, Computed tomography.

A 59-year-old female patient with an unremarkable medical history was referred to our hospital with contained innominate artery rupture and a large mediastinal mass. Initially, she had presented to an external hospital with acute progression of severe back pain. The patient had no evidence of neurologic disorders and no fever. Inflammation markers were elevated, ie, C-reactive protein at 310.2 mg/L, leucocytes were 31.47 nL−^1^ and procalcitonin was 0.30 ng/mL. Computed tomography (CT) scanning showed a large mass in the anterior mediastinum (Figure 1, *A*) and a contained rupture of an innominate artery aneurysm close to the aortic arch, measuring 14 mm × 22 mm × 13 mm (Figure 1, *B* and *C*). The patient was referred to our cardiac surgery department, in stable hemodynamic condition, to undergo emergent surgery.

The right femoral vessels were surgically exposed and cannulated. After heparinization, cardiopulmonary bypass was commenced, and cooling to a target temperature of 24°C was initiated. The next step was to expose and cannulate the right axillary artery through an infraclavicular incision. Thus, double arterial cannulation was established. After median sternotomy, a large amount of pus began to escape the mediastinum, and microbiologic specimens were collected. Severe mediastinal adhesions then became apparent. During preparation, severe bleeding was encountered. As the body temperature had already reached 24°C, a brief period of circulatory arrest was initiated for 8 minutes, to allow for preparation of the ruptured site. The innominate artery and the adherent part of the aortic arch were ruptured. The bleeding site was controlled by distal clamping of the innominate artery and corresponding partial clamping of the aortic arch. Circulation was reinitiated and after crossclamping, cardioplegic arrest was obtained by antegrade application of 1500 mL of Bretschneider cardioplegia. After opening of the aortic arch, a balloon-tipped perfusion catheter was introduced into the left common carotid artery so that bilateral antegrade cerebral perfusion at a rate of 10 mL/kg/min was provided. The defect in the aortic arch was resected. Aortic arch reconstruction was performed using a patch of bovine pericardium. Cardiopulmonary bypass was reinitiated, rewarming was started, and the aortic crossclamp was removed. Further resection and debridement of the innominate artery was required. A 10-mm vascular Dacron graft was soaked in rifampicin and sutured to the distal end of the innominate artery, using a running 4-0 polypropylene suture. The proximal aortic arch was partially clamped again, and the vascular graft was sutured to the aortic pericardial patch in an end-to-side fashion, using a running 4-0 polypropylene suture. After rewarming and reperfusion, cardiopulmonary bypass was weaned. Sternal closure and wound closures were performed in standard fashion. The total circulatory arrest time was 8 minutes for identification and preparation of the rupture site, plus 45 minutes for arch reconstruction on antegrade cerebral perfusion. The total procedure time was 310 minutes (Figure 1, *D*).

The patient was transferred to the intensive care unit. One episode of generalized seizure on postoperative day 1 was treated with levetiracetam. Further CT scans were performed and showed no sign of intracranial hemorrhage or stroke. The patient was extubated on postoperative day 2. *S aureus* was detected in intraoperative specimens and blood cultures. Antibiotic treatment with flucloxacillin and fosfomycin was continued for 6 weeks. She recovered without further issues. One year later, she was doing well without any evidence of neurologic disorders. Going forward, CT or magnetic resonance imaging scans at regular intervals, similar to aortic dissection cases, are recommended.

## Discussion

Mycotic aneurysm of the innominate artery is an extremely rare condition. Several approaches have been described for aneurysms in this location.^1^ Our strategy for this emergent case was double arterial cannulation of the femoral artery and the axillary artery. It allowed for evenly distributed cooling of the patient. Preparation of the aortic arch and the innominate artery was performed at 24°C, allowing for brief episodes of total circulatory arrest in the event of bleeding providing a dry field but avoiding complications of deeper hypothermia. After gaining control of either side of the ruptured aneurysm by clamping, partial perfusion could then be reinitiated. This is a further applicable and likely underrated concept,^2^ also usable in aortic dissection or thoracoabdominal aortic repair. Double arterial cannulation also had the advantage in that no balloon-tipped perfusion catheter needed to be advanced through the infected and severely damaged innominate artery to provide right-sided antegrade cerebral perfusion. Furthermore, cardiopulmonary bypass could be reinitiated together with full rewarming, once the aortic arch was repaired.

Different strategies for replacement of the infected aneurysm have been reported, such the use of a venous graft,^3^ bovine pericardium,^4^ or a rifampicin soaked Dacron graft.^5^ A patch of bovine pericardium was used to repair the aortic arch defect, because it was the simplest and fastest solution to address the aortic arch pathology. It also allowed us to minimize circulatory arrest time. As for the innominate artery, we chose a rifampicin-soaked Dacron graft over other options in this case because it was the quickest solution in this urgent scenario. After antibiotic treatment for 6 weeks, a favorable outcome was achieved with our approach.

In summary, this case demonstrates that prudent cannulation strategies allow for successful treatment of rare and complex pathologies, such as ruptured mycotic aneurysm of the innominate artery. Written informed consent for publication was obtained. Institutional review board approval is not required for case reports at our institution.

## Conflict of Interest Statement

The authors reported no conflicts of interest.

The *Journal* policy requires editors and reviewers to disclose conflicts of interest and to decline handling or reviewing manuscripts for which they may have a conflict of interest. The editors and reviewers of this article have no conflicts of interest.
